# Risk of relationship separation in men with Peyronie’s disease in a matched Swedish cohort

**DOI:** 10.1038/s41598-024-72390-3

**Published:** 2024-09-10

**Authors:** Lars Henningsohn, Henrik Larsson, Ralf Kuja-Halkola, Martin Cederlöf

**Affiliations:** 1 Department of Clinical Science, Intervention, and Technology (CLINTEC), Karolinska Institutet, Stockholm, Sweden; 2 Faculty of Medicine and Health, School of Medical Sciences, Örebro University, Södra Grev Rosengatan 30, 70362 Örebro, Sweden; 3 Department of Medical Epidemiology and Biostatistics (Solna), Karolinska Institutet, Stockholm, Sweden

**Keywords:** Peyronie’s disease, Penile curvature, Divorce, Relationship separation, Urogenital diseases, Psychology

## Abstract

Peyronie’s disease (PD) has detrimental effects on the quality of life, mental health, sexual functioning and several other aspects that increase the risk of relationship problems. However, no study to date has assessed the risk of relationship separation in med with PD. Herein, we utilized data from Swedish national registers to examine the risk of relationship separation in men with PD. We conducted a matched cohort study on men born 1933–1992, followed from 1997 to 2013. PD was defined as a physician-assigned diagnosis according to the International Classification of Diseases, Tenth version. Each man with PD (n = 8020) was matched with 10 comparison men. We defined relationship separation as (1) ever separated, and (2) separation rate. We used log-linear regression to estimate the risk ratio, and rate ratio of relationship separation. We adjusted for matching variables (birth year and country of birth), and an indicator of each follow-up year. We found that men with PD had a 13% increased risk of relationship separation (risk ratio 1.13, 95% confidence interval [CI] 1.08–1.17). The rate of relationship separation events, measured on a yearly basis, was increased by 18% (rate ratio 1.18, CI 1.12–1.24), and remained similar when adjusting for follow-up year and socio-economic status.

## Introduction

Peyronie’s disease (PD) is a connective tissue disorder that affects the tunica albuginea of the penis, and results in a build-up of fibrotic tissue (“plaques”) that causes penile curvature, shortening, atypical shapes during erection, erectile pain, and erectile dysfunction^1^. Although PD was described by Francois Gigot de la Peyronie in 1743^2^, the etiology remains poorly understood. Proposed risk factors include genetic susceptibility, micro trauma, pro-inflammatory processes, and abnormal wound healing^2,3^. The prevalence is uncertain, but has been estimated to 3–9%^4,5^. However, in a population-based study from the US based on self-reports, the prevalence was estimated in two groups; patients reporting symptoms only; and patients reporting diagnosis, symptoms, or treatment. In the latter group, the prevalence was observed to be 13.1%^6^.

PD is associated with substantial suffering, and negative effects on the quality of life^7^, mental health^7–9^, self-esteem, and self-confidence^7^. In addition, distress, psychological problems, emotional problems, and a distorted self-image are observed in a majority of men with PD—problems that are rarely brought up in the consultation and therefore often goes untreated. For these reasons, PD is very challenging to live with^7,8^. Furthermore, In a recent study from our group, we assessed the psychiatric burden of PD in a large sample, and observed excess risks of between 40 and 100% for diagnosed depression, anxiety, substance use disorder, and self-injurious behaviors^9^.

The complex problem profile many men with PD display is likely associated with serious relationship problems, but research into this topic is limited. In one study by Smith et al.^10^ 54% reported relationship problems. Further insights has been provided from thematic discussions on internet group boards directed to men with PD, who described problems related to relationship functioning, such as avoidance of intimacy, fear of rejection, feelings of isolation, and fear of dating^11^. Moreover, sexual problems is an important aspect of relationship functioning, and problems with sexual function and performance frequently occur in men with PD^7,8^. In an influential qualitative study^12^, PD patients identified such problems as a core domain. Taken together, more research into relationship problems in men with PD is needed. As relationship separation entail excess risks of life-threatening outcomes such as suicide^13^, the absence of research on this outcome represents a particularly important knowledge gap.

The aim of this study was to assess the risk and rate of relationship separation in men with PD, as compared with men without PD. To accomplish this, we employed a large, nationwide sample of Swedish men, and utilized data from Swedish national registers. Ethical approval was obtained from the Swedish Ethics Review Authority with DNR 2013/862-31/5. Informed consent was deemed unnecessary according to the Swedish Ethics Review Authority.

## Methods

### Participants

**Fig. 1:**
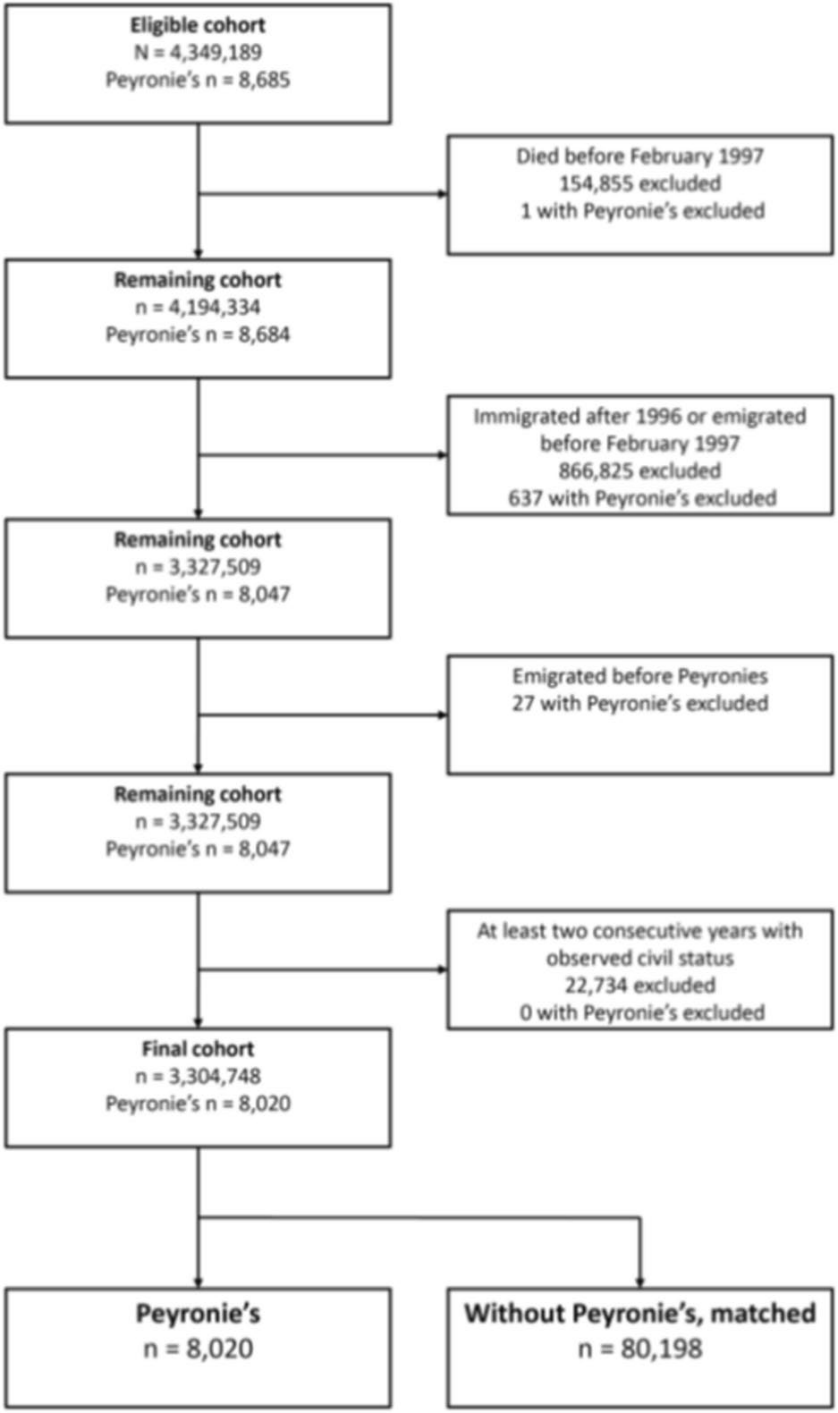
Flowchart of the selection of the matched cohort.

Using the Total Population Register^14^ we identified all men born between 1933 and 1992 (N = 4,349,189). Figure 1 shows the number of individuals excluded by different criteria; death or emigration before February 1997 or immigrated after 1996, PD diagnosis after emigration, and less than two consecutive years with observed civil status (see below for details). The resulting analytic cohort comprised 3,304,748 individuals, of whom 8020 (0.2%) had PD. We followed the study participants until December the 31st 2013.

### The matched cohort procedure

From the identified analytic cohort, we individually matched each index person with PD with up to 10 men without PD on the variables birth year and country of birth (in region categories, see below for details). Additionally, each matched individual had to be alive and in Sweden at the date of the PD diagnosis of the index person they were matched with. The matching was successful for all but one single man, who was matched with 8 men without PD instead of 10, resulting in 8020 men with PD and 80,198 men without PD; in total 88,218 men.

### Analytic variables and registers

PD was exposure of the study and defined as a diagnosis of the disorder in the National Patient Register^15^ (N48.6), and assigned by the treating physician according to the International Classification of Diseases, 10th version (ICD-10)^16^. The ICD-10 was introduced in Sweden 1997. In the analyses, PD diagnoses were treated both as a binary ever/never variable and as a time-varying variable, where men were considered exposed from the year they got the diagnosis, and unexposed prior to that year.

We used the Longitudinal integrated database for health insurance and labor market studies (LISA)^17^ to identify outcome variables such as civil status (unmarried, married, registered partner, divorced, ended registered partnership, widower, or surviving registered partner) for the available years (1990–2013). The civil status measurement was based on official records of registered marriage and partnership, and whether registered marriage and partnership had ended, by December 31st each year. We did not separate opposite sex and same sex marriage and partnership. We then collapsed information on marriage/registered partnership into one variable, below referred to as civil status, and we hereafter refer to the ending of a marriage or registered partnership as a relationship separation.

We constructed two outcome variables using the observed civil status:

1. *Ever separated *Defined as at least one observed divorce status (note that this separation could have happened before 1990).
2. *Separation rate* For each year between 1991 and 2013, a separation was observed if civil status went from being partnered the year before to being separated the concurrent year.

*Covariates* Matching variables were birth year, and country of birth categorized into regions according to Statistics Sweden: Sweden, Nordic countries but Sweden, EU28 but Nordic countries, Europe but EU28 and Nordic countries, Africa, North America, South America, Asia, Oceania, Soviet Union. We used LISA to identify variables used as proxies for socioeconomic status; education and income. For this we used the years 1990–1996 and took the highest observed education, categorized as ≤ 9 years, 10–12 years, and > 12 years, and the highest observed income, in quantiles per year among the individuals included in the analyses. Furthermore, since separation event was evaluated each year, we utilized observation year as a covariate.

### Statistical analyses

*Ever separated* In the analysis of this outcome measure, we used log-linear regression to estimate the risk ratio of relationship separation. To adjust for matching variables, we used conditional generalized estimating equation regression^18^, and conditioned on the matching clusters. As the *ever separated* outcome could have happened at any time, including prior to 1990, we only considered PD as a binary ever/never exposure. We fitted a model only adjusted for matching variables, a model where the outcome was weighted for number of years with observed civil status, and a model further adjusting for socioeconomic status.

*Separation rate* We used log-linear regression to estimate the rate ratio of relationship separation per year of follow-up, again conditioning on matching cluster in a conditional generalized estimating equation model. First, we analyzed the association by treating PD as a binary ever/never exposure, using all follow-up years; 1991–2013. Second, we utilized the year of PD diagnosis to treat PD as a time-varying exposure during years when ICD-10 was implemented in Sweden (1997–2013), considering a man exposed since the year of the PD diagnosis. For each exposure definition, we fitted a model adjusted only for matching variables, a model additionally adjusted for an indicator for each follow-up year, and a model further adjusting for socioeconomic status. Finally, we ran within-individual analyses in men with PD diagnoses, comparing years before diagnosis with years after regarding relationship separation events, conditioning on the identifier of each specific man. This analysis adjusts for all constant factors within each person, such as birth year, birthplace, genetics, and socioeconomic background; however, it does not adjust for time-varying factors such as ageing. We used the statistical software’s SAS and R for data management, and R for statistical analyses. The conditional generalized estimating equation models were fitted using the library “drgee”^19^. Missingness in socioeconomic variables (5.4% had any missing value) were handled using listwise deletion.

### Sensitivity analyses

We performed three sensitivity analyses. First, since we did not know whether the PD diagnosis or relationship separation happened first, we re-ran analyses with time-varying PD exposure analyses assuming that the men were exposed starting the year after observed PD diagnosis. Second, to investigate whether relationship separation happened very close to a potential PD diagnosis, we re-ran the time-varying exposure analyses assuming only the year of PD diagnosis was exposed. Third, we also performed these analyses adjusting for socioeconomic status. Finally, we performed within-individual analysis for these two additional time-varying definitions of exposure to PD. All methods were carried out in accordance with relevant guidelines and regulations.

## Results

In Table 1 we present descriptive information. The experience of ever having separated was more common in men with PD (23.8%) than in the matched comparison group (21.1%). The mean count of separation events during the follow-up years was also higher among men with PD, compared with men without PD (0.18 *versus* 0.15). Men with and without PD had similar mean follow-up time, and the matching variables were perfectly balanced.

**Table 1:** Peyronie’s disease status, by relationship separation measurements, and birth year.

	Men with PD	Men without PD
N (%)	8020 (9.1%)	80,198 (90.9%)
Ever separated, n (%)	1907 (23.8%)	16,928 (21.1%)
Separation count, mean (SD) [range]^a^	0.18 (0.43) [0,4]	0.15 (0.39) [0,5]
Follow-up years, mean (SD) [range]^b^	23.1 (2.8) [4,24]	23.0 (2.9) [3,24]
Education		
≤ 9 years	2083 (26.0%)	22,189 (27.7%)
10–12 years	3572 (44.5%)	35,133 (43.8%)
> 12 years	1943 (24.2%)	18,518 (23.1%)
Missing	422 (5.3%)	4358 (5.4%)
Income		
1st quintile	437 (5.4%)	6065 (7.6%)
2nd quintile	1031 (12.9%)	11,450 (14.3%)
3rd quintile	1250 (15.6%)	12,290 (15.3%)
4th quintile	1795 (22.4%)	17,499 (21.8%)
5th quintile	3147 (39.2%)	29,294 (36.5%)
Missing	360 (4.5%)	3600 (4.5%)
Foreign born, n (%)^c^	928 (11.6%)	9278 (11.6%)
Birth year 1933–1942, n (%)	1454 (18.1%)	14,540 (18.1%)
Birth year 1943–1952, n (%)	2815 (35.1%)	28,148 (35.1%)
Birth year 1953–1962, n (%)	1788 (22.3%)	17,880 (22.3%)
Birth year 1963–1972, n (%)	1070 (13.3%)	10,700 (13.3%)
Birth year 1973–1982, n (%)	616 (7.7%)	6160 (7.7%)
Birth year 1983–1992, n (%)	277 (3.5%)	2770 (3.5%)

^a^For the years 1991–2013.

^b^For the years 1990–2013.

^c^Note that men were matched on grouped countries of birth.

### Results from the ever separated analyses

Men with PD were 13% more likely to ever have experienced a relationship separation, than men without PD (risk ratio 1.13, 95% confidence interval [CI] 1.08–1.17; Table 2). This association remained similar when weighting the risk by available follow-up time (risk ratio 1.12, CI 1.08–1.17), as well as when adjusting for socioeconomic status (risk ratio 1.14, CI 1.09–1.19).

**Table 2:** Results from the regression analyses. Estimates are risk ratios and rate ratios, and corresponding 95% confidence intervals.

	Ever separated^a^	Time-constant PD, separation rate^b^	Time-varying PD, separation rate^c^
Peyronie’s	1907/8020; 23.8%	1454/176,913; 8.21 separations per 1000 person-years	338/54,554; 6.20 separations per 1000 person-years
Not Peyronie’s	16,928/80,198; 21.1%	12,325/1,762,668; 6.99 separations per 1000 person-years	9123/1,398,105; 6.53 separations per 1000 person-years
Adjusted for matching variables^d^	1.13 (1.08–1.17)	1.18 (1.12–1.24)	1.03 (0.93–1.15)
Additionally adjusted for each year^e^	1.12 (1.08–1.17)	1.17 (1.11–1.23)	1.18 (1.06–1.32)
Additionally adjusted for socioeconomic status	1.14 (1.09–1.19)	1.16 (1.10–1.23)	1.16 (1.03–1.30)

N/A, not applicable.

^a^Risk ratio of being separated once.

^b^Rate ratio of separations per year of follow-up, years 1991–2013.

^c^Rate ratio of separations per year of follow-up, with PD diagnoses treated as a time-varying variable, years 1991–2013.

^d^Adjusted for matching on birth year and birth region. For within-individual analysis, adjusted for all constant factors within each man.

^e^For the “ever separated” outcome was additionally weighted by years of follow-up. For “separation per year” outcome, the analyses were additionally adjusted for each year’s baseline rate of separations.

### Results from the separation rate analyses

All results from these analyses are presented in Table 2. When PD was treated as a binary ever/never variable, an 18% increased rate of relationship separation (rate ratio 1.18, CI 1.12–1.24), was observed in men with PD, and remained similar when adjusting for rates during each follow-up year (rate ratio 1.17, CI 1.11–1.23), and did not change meaningfully when further adjusting for socioeconomic status (rate ratio 1.16, CI 1.10–1.23).

When we incorporated the PD diagnosis date and considered men as exposed to PD from the year of diagnosis (and unexposed before), the risk of relationship separation was attenuated and statistically insignificant (rate ratio 1.03, CI 0.93–1.15). When yearly rates of separation were additionally adjusted for, the risk estimate increased to a similar level as in the “time-constant PD” analysis (rate ratio 1.18, CI 1.06–1.32), also when further adjusting for socioeconomic status (rate ratio 1.16, CI 1.03–1.30).

### Sensitivity analyses

First, when alternative exposure period definitions were assessed, similar results as in the main analysis when exposure started year after PD diagnosis was observed, but weaker and with a statistically non-significant association when exposure was taken to be only the year of PD diagnosis (Table 3). Second, adjustment for socioeconomic status for these alternative exposure definitions did not yield changes in general inference (Table 3). Finally, the within-individual analysis yielded results in the opposite direction; the odds of separation was increased by 17%after a PD diagnosis compared to before a PD diagnosis (rate ratio 0.83, CI 0.71–0.96; Table 3); the result was statistically non-significant when exposure was defined as only the year of PD diagnosis.

**Table 3:** Sensitivity analyses expressing the effects of different definitions of exposure to PD diagnosis and estimates for within-individual analyses.

	Exposed from year of PD diagnosis	Exposed from year after PD diagnosis	Exposed only year of PD diagnosis
Peyronie’s	338/54,554; 6.20 separations per 1000 person-years	286/46,589; 6.14 separations per 1000 person-years	52/7965; 6.53 separations per 1000 person-years
Not Peyronie’s	9123/1,398,105; 6.53 separations per 1000 person-years	9175/1,406,070; 6.53 separations per 1000 person-years	9409/1,444,694; 6.51 separations per 1000 person-years
Time-varying PD diagnosis, separation per year^b^	1.18 (1.06–1.32)^a^	1.20 (1.07–1.36)	1.07 (0.81–1.40)
Time-varying PD diagnosis, separation per year, adjusted for socioeconomic status^b^	1.16 (1.03–1.30)^a^	1.16 (1.02–1.32)	1.11 (0.83–1.48)
Within-individual^c^	0.83 (0.71–0.96)	0.85 (0.72–0.99)	0.87 (0.66–1.15)

^a^Result equal to that found in Table 2; presented here for comparison purposes.

^b^Adjusted for matching on birth year and birth region, additionally adjusted for each year’s baseline rate of separations.

^c^By design adjusted for all variables constant within the individuals.

Estimates (risk ratios and rate ratios) and 95% confidence intervals.

## Discussion

In this nationwide, matched cohort study based on Swedish national registers, we examined the risk of two measures of relationship separation in men with PD, compared with a comparison group of matched men without PD randomly selected from the general population. To the best of our knowledge, this was the first study to assess this research question, which is an important gap in current knowledge.

We observed that men with PD had a 13% elevated risk of ever having experienced a relationship separation, and a rate ratio increment of 18% of experiencing a relationship separation when PD was treated as a binary phenomenon. These crude results remained unchanged in the adjusted analyses. In the time-varying analyses, the rate ratio of relationship separation was attenuated to approximately 1, but increased corresponding to a rate ratio increment of 18% when yearly relationship separation rates were adjusted for.

Socioeconomic status did not seem to affect the results in any meaningful way.

In the sensitivity analyses, similar results were observed. When we performed within-individual analyses using different time-varying PD definitions, the association was in the opposite direction.

These findings are in general consistent with the limited number of previous reports of relationship problems in men with PD^11^, and represents a decrease of an important gap in current knowledge by, for the first time, showing that PD index excess risks of relationship separation. Relationship separations are serious events; in a seminal paper, Kposowa^13^ reported a more than doubled risk of suicide in separated individuals, compared with married individuals (risk ratio 2.08, 95% CI 1.58–2.72). There was also a tendency towards an even higher suicide risk among men (risk ratio 2.38, 95% CI 1.77–3.20). Moreover, relationship separation additionally associates with a range of pan-domain adversities, including negative short, and long-term effects on economy^20^, psychological adjustment^21^, physical health^21,22^, quality of life^21^, and life expectancy^22^. Thus, relationship separation is an important marker of adverse, and even life-threatening consequences.

One could argue that since a majority of men with PD suffer from for example severe distress^7^, psychological problems^7,8^, emotional problems^7,8^ and in addition present excess risks of a range of mental disorders,^9^ are prone to end up in a particularly vulnerable situation after a relationship separation with surplus risks of negative consequences, although data that supports this notion are currently lacking.

A probable explanation for the attenuated rate ratio of relationship separation observed in the time-varying analysis, which was substantially increased when yearly rates of relationship separation was adjusted for, is that the likelihood of relationship separation risk decreases by increasing age.

The finding of a decreased risk of relationship after a PD diagnosis, compared with before diagnosis from the within-individual analysis was intriguing. A possible explanation could be that men engaged in family planning who experience sexual problems that may hinder intercourse seek help for their problems, for example at family planning units, where they subsequently receive some form of successful intervention, psychosocial support, or counseling that mitigates the risk of a relationship separation**.** Another possibility is that, similarly as above, the likelihood of relationship separation risk decreases by increasing age. Taken together, the current study demonstrated that men with PD display an increased risk of experiencing a relationship separation, which highlights the need for health care to routinely offer partner counseling to men with PD. Of note, an important finding in a study by Rosen et al.^12^ was that none of the participating men with PD had sought help together with their partner, suggesting that practitioners should encourage men with PD to bring their partners to their visits at health care- and relationship counseling units.

### Implications for primary care of men with PD

As primary care is the first system most men will visit first after the onset of their disorder, the primary care will have the continued responsibility of the patient in many countries. Due to the complex problem profile many men with PD exhibit, including an over-representation of major mental disorders and self-injurious behaviors, we have previously stated that all men with PD should have a documented mental health status, under the responsibility of primary care, and preferably performed at the first visit^9^. According to our assessment, primary care should also be the health care system with the responsibility to ensure that all units will either refer men with PD and their partners to relationship counselors, or make sure to inform about other available counseling options, and that this should apply every new PD patient who are in a relationship at their first visit. Many of the problems men with PD experience, such as the psychological burden, do not decline over time^7^. This is likely a consequence of a lack of appropriate and well-timed treatment. To improve the care of men with PD, and potentially reduce the risk of negative consequences, in this case relationship separation, we stress the importance of the appropriate actions already at the first contact with primary care. A survey from 2007 about the knowledge of PD among American primary care physicians and urologists revealed limited knowledge and incorrect assumptions of the disorder^23^. Although these findings may not apply today, or to other countries, our recommendations require a high degree of awareness of PD and its complex problem profile among physicians in primary care.

### Implications for specialist care of men with PD

Although primary care typically “own” the care pf PD patients, urologist should also consider the relevance of the findings in the current study for their clinical practice. Based on the results observed herein, we strongly suggest that urologists should ask their PD patients about their relationship status and potential relationship problems.

### Strengths and limitations

This study has strengths; it is based on a large, nationwide sample of men with physician-assigned PD diagnoses along with matched comparison subjects; the measurements of separation relied on a solid data source, and the statistical analyses were performed with state-of-the-art methods. This study also has several limitations that should be considered; the study included only help-seeking men with PD, which may have introduced bias resulting in deflated or inflated the estimates. Research based on self-reported PD is warranted to elucidate this important issue. Moreover, our data does not allow for causal interpretations, and we did not have complete follow-up for the full cohort.

## Conclusions

For the first time, we have showed that men with PD had 13% increased risk-, and an 18% elevated rate, of relationship separation. These findings decrease an important gap in current knowledge, and have implications for health care and reseach.

## Data Availability

The data that support the findings of this study are available from Statistics Sweden, but restrictions apply to the availability of these data, which were used under license for the current study, and so are not publicly available. Data are however available from the authors upon reasonable request and with permission of Statistics Sweden. For any requests regarding data availability, contact the lead author.
